# Spindle Cell Lipoma of the Shoulder: A Case Report

**DOI:** 10.7759/cureus.41961

**Published:** 2023-07-16

**Authors:** Saleh A AlSuwaydani, Mohammed Alfehaid, Abdulaziz S Alshamikh, Waleed A Almaiman

**Affiliations:** 1 Department of Surgery, Unaizah College of Medicine and Medical Sciences, Qassim University, Unaizah, SAU; 2 Department of Emergency Medicine, Unaizah College of Medicine and Medical Sciences, Qassim University, Unaizah, SAU

**Keywords:** kingdom of saudi arabia (ksa), alqassim region, shoulder lipoma, spindle cell lipoma, lipoma, general surgery

## Abstract

Spindle cell lipoma (SCL), an uncommon variant of benign lipomatous tumor, occurs predominantly in the posterior neck or the shoulders. Patients usually present a well-circumscribed and non-aggressive subcutaneous mass. Histopathologically, the mass comprises fat, CD34-positive spindle cells, and ropey collagen on a myxoid matrix. We report a case of left-shoulder SCL in a 45-year-old Saudi male nonsmoker with no known trauma. A soft tissue mass was seen on MRI near the posterior shoulder. It was a 4.5 cm x 3 cm subcutaneous rounded lump with heterogeneous signal intensity. There is no aggressive or invasive feature, and there is no significant fatty content. The mass was surgically excised with no complication. Histological examination revealed a neoplastic lesion constituted of mature adipocyte lobules and proliferation of loosely held bland spindle-shaped cells against a myxoid background. These cells exhibit neither increased mitoses nor nuclear pleomorphism. There were intervening rope collagen bundles. Lipoblasts and atypical stromal cells are also unidentified. At the periphery, a thin capsule was identified. There is no evidence of malignancy. To the best of our knowledge, this is the first reported case in Unaizah, Al Qassim. The correct diagnosis of SCL without a lipogenic component may be made by paying close attention to the clinical setting, histologic characteristics, immunohistochemical profile, and chromosomal abnormalities.

## Introduction

Spindle cell lipoma (SCL) is a rare, slow-growing, benign lipoma that was initially documented in 1975 [1-4]. The composition of SCL consists of mature adipocytes, spindle cells that proliferate uniformly and are located beneath a collagen stroma resembling a rope, as well as a mucoid stroma. SCL is found in approximately 1.5% of lipomas that have been analyzed through biopsy [1-6]. SCL frequently manifests as a distinct and varied subcutaneous mass. The prevalence of this condition is highest among individuals in the middle-aged male population due to the expression of androgen receptors by SCLs, with a male-to-female ratio of 10:1 within the age range of 45 to 70 years. Furthermore, it exhibits a particular inclination toward affecting the “shawl region,” which encompasses the shoulder, posterior neck, and upper back [2,7]. While the histological examination is widely considered the most reliable method for diagnosis, it is worth noting that clinical presentation and imaging techniques can offer valuable insights for a more precise preoperative assessment. This can greatly assist surgeons in making informed decisions regarding the need for biopsy, resection, or observation [8].

## Case presentation

History and physical examination

A 45-year-old Saudi man, a nonsmoker with no known medical issues presented a left shoulder swelling that had been increasing without pain throughout a five-year history. The patient, who had no prior history of trauma, noticed the growth by chance. As the swelling grew in size, it became uncomfortable for the patient to wear clothes and drew the attention of others at work and the gym. Lipoma was suggested during the initial investigation. An evaluation of symptoms found that B-symptoms, such as fever, weight loss, and night sweats, were not present. And no family history is present.

The patient seemed to be in good health and was cooperative throughout the examination. He was conscious and oriented to time, place, and person. His vital signs were normal, and his neurovascular examination was unimpaired. During the examination of the shoulder, there was a fixed shoulder mass that measured 6 cm by 6 cm: mainly due to his thick skin. This mass was not moving in relation to dimensions and did not pose any restrictions on the patient's range of motion at the shoulder joint. The mass was round in shape with ill-defined borders and an irregular surface. When palpated, the swelling had the consistency of a firm mass. It was on a subcutaneous plane, with the overlying skin remaining undamaged and detached. There was no indication of lymphadenopathy in the regional area. Clinical evidence points to the presence of a lipoma.

Pre-operative investigation

**Figure 1:**
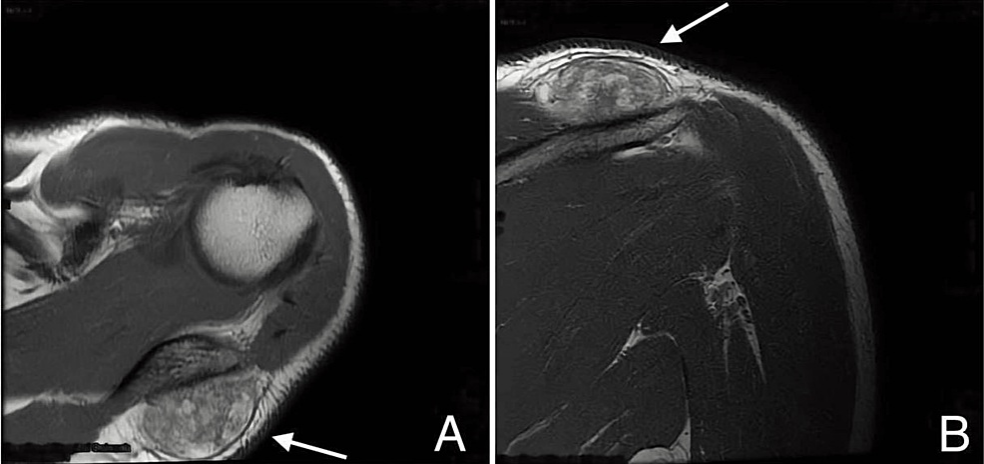
T1 MRI of the shoulder (A) Axial view.(B) Coronal view.

The pre-operative MRI revealed the presence of a soft tissue mass near the posterior shoulder. It was a subcutaneous lump that measured 4.5 centimeters long by 3.5 centimeters wide by 2 centimeters depth and had a round shape. The signal intensity was heterogeneous. There was no aggressive nor invasive feature, nor was there a substantial amount of fatty content seen (Figures 1A, 1B).

Procedure and follow up

After the risks and benefits of the surgical excision were discussed, a patient's informed consent was obtained for an elective procedure to excise the mass. At first, the area surrounding the skin lesion was prepared and draped in the usual sterile manner and then was anesthetized. Skin incision was made in a linear fashion and lipoma was excised bluntly using small forceps. Hemostasis was assured. The mass was submitted to histopathology for examination under a microscope measuring 5 cm long by 4 cm wide by 2 cm high. After receiving confirmation from the histopathology that the sample did not contain any features indicative of malignancy, the incision was irrigated and then closed in layers. The patient tolerated the procedure well. The patient was discharged one day after the operation, the dressing was changed upon discharge under sterile technique, and six weeks later, when the patient was followed up on, there were no more complaints, the wound was healing, there was no discharge, and there were no new masses.

Post-operative investigations

**Figure 2:**
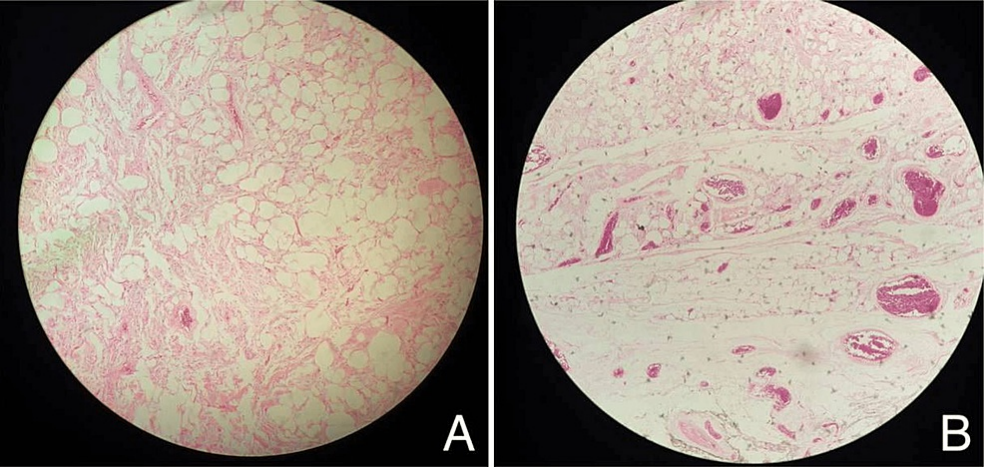
Microscopic picture of Spindle Cell Lipoma (A&B)

Post-operative microscopic description revealed a neoplastic lesion composed of an admixture of lobules of mature adipocytes and proliferation of loosely held bland spindle-shaped cells against myxoid background. These cells do not show increased mitoses nor nuclear pleomorphism. Intervening rope collagen bundles were seen. Atypical stromal cells and lipoblasts are also not identified. A thin capsule was focally identified at the periphery. There is no evidence of malignancy (Figures 2A, 2B).

A panel of immunohistochemical stains is performed, and the following reactivity pattern is observed in spindle cells: CD34: Positive; SMA: Positive; Desmin: Negative; and S100: Non-contributory.

## Discussion

A common subcutaneous tumor, known as a lipoma, is made up of adipose (fat) cells and is often surrounded by a thin layer of fibrous tissue. Lipomas may occur in both adults and children. In point of fact, these neoplasms make up the majority of those seen by medical professionals. Patients often have symptoms in the cephalic area of the body, particularly in the head, neck, shoulders, and backs of their bodies, according to clinical observations. The subcutaneous tissues of patients are the most prevalent location for tumors to be discovered. Even though the ages at which the masses emerge might vary, doctors almost never need to treat them since they are almost always benign. They are not hazardous to the patient unless they are uncomfortable owing to their position on joints or are developing at an excessive pace, both of which are uncommon for lipomas due to the slow growth rate that is characteristic of lipomas. One of the numerous unusual types of lipomas, which may take many different shapes, is known as SCL [9].

In 1975, Enzinger and Harvey were the ones who made the first discovery of SCL [1]. It most often manifests itself in the subcutaneous tissues of the neck, shoulder, or back, and it typically strikes males between the ages of 45 and 65 [2]. There is still a lot of mystery surrounding the pathophysiology of SCL. A number of different cell types, including fibroblasts, adipocytes, immature mesenchymal cells, and CD34-positive dendritic interstitial cells, have been postulated to be the precursors of spindle cells [1,2].

The lesion that may be seen inside a myxoidstroma is made up of mature adipocytes, very small homogeneous spindle cells, and eosinophilic collagen bundles. According to immunohistochemical labeling, spindle cells are positive for CD34 but negative for S-100 protein. This is in contrast to lipoma cells, which usually only include an adipocyte component and generate a lobular pattern that is embedded in a collagenous matrix. On the other hand, CD34 is not a marker that is only seen in SCLs; rather, it is a signal that may stain positively for various cancers that fall within the lipoma differential diagnostic range. Desmin negativity as well as chromosomal deletions on 12q and/or 16q in SCLs may be helpful in making the diagnosis [10].

The differential diagnosis may include a variety of benign lipomatous tumors made up of mature adipocytes with or without additional components of mesenchymal tissue. Another item that has to be separated is well-differentiated liposarcoma (WDL), which is the most important differential diagnosis. In regions that are analogous to SCL and have boundaries that are difficult to identify, WDL is commonly encountered. Through meticulous histological examination, the features of liposarcoma invasion may be identified. The uniformity of the spindle cell shape, in addition to the lack of lipoblasts, which are not present in SCL, will tip the scales in favor of SCL. It is possible that liposarcoma is present when a “lipoma” that has been excised in the past continues to come back. In contrast to spindle cell lipoma, liposarcoma demands significant broad excision. As a direct consequence of this, we were able to rule out WDL as well as other malignancies. Because WDL very seldom includes spindle-shaped cells that are positive for CD34, the presence of the tumor provided additional evidence for our diagnosis of SCL [11].

SCLs that are fat-free or have a low amount of fat are at one end of a vast range of imaging characteristics created by changes in the ratio of fat to spindle cells. These lipomas are difficult to diagnose with radiological scans because of their overlap and similarities with other soft tissue tumors. In the case that was given, magnetic resonance imaging (MRI) showed that there was a soft tissue mass at the posterior shoulder that was sized 4.5 centimeters by 3 centimeters and had a round appearance beneath the skin. There have not been any invasive or aggressive behaviors, and there was not a considerable amount of fatty content. The advanced age of the patient, the lack of B symptoms, and an intraoperative assessment of a multilobulated fatty projecting mass were all indicators that the patient had a lipoma. Fortunately, a pathological examination determined that the tumor was an SCL, which may be easily treated by removing the affected tissue via an excision.

## Conclusions

In conclusion, a lipoma with a spindle cell subtype may provide a diagnostic difficulty to radiologists, pathologists, and surgeons since it often presents as a lesion with little or no macroscopic or microscopic fat. SCLs may also have intramuscular extension. Due to the radiographic overlap and similarities with liposarcoma, schwannoma, and other soft tissue tumors in the presence of these characteristics, imaging-based identification of SCLs may be particularly challenging, biopsy followed by surgical removal is suggested. The imaging features of SCLs in the US, CT, PET CT, and MRI may enable the radiologist to suggest this diagnosis prior to biopsy or excision, allowing the physician to avoid unduly aggressive procedures that may result in higher postoperative morbidity.
